# Commentary: Probing Genomic Aspects of the Multi-Host Pathogen *Clostridium perfringens* Reveals Significant Pangenome Diversity, and a Diverse Array of Virulence Factors

**DOI:** 10.3389/fmicb.2018.01856

**Published:** 2018-08-14

**Authors:** Iman Mehdizadeh Gohari, John F. Prescott

**Affiliations:** Department of Pathobiology, University of Guelph, Guelph, ON, Canada

**Keywords:** *Clostridium perfringens*, whole genome sequencing, comparative genomics, genetic relatedness, genetic variation, pangenome, clostridial infection

We recently read with great interest the paper entitled “Probing Genomic Aspects of the Multi-Host Pathogen *Clostridium perfringens* Reveals Significant Pangenome Diversity, and a Diverse Array of Virulence Factors” by Kiu et al., Front. Microbiol., December 2017.

In the study, comparative genomic analyses and genetic relatedness of 56 *Clostridium perfringens* (including 51 genomes available in GenBank and 5 *C. pefringens* strains sequenced by these researchers) was assessed using various bioinformatic approaches (Kiu et al., 2017). Although ~60% of sequences are from a single pathotype (*netF*-positive isolates), the work has contributed significantly to understanding of genomic diversity of this bacterium. However, there is a clear mistake in the “*in silico* profiling of virulence traits” analysis. Kiu et al. noted that “*netB, netE, netF*, and *netG* genes were found to be uniquely encoded within JFP isolates (associated with haemorrhagic enteritis in dogs and foals), as reported previously (Mehdizadeh Gohari et al., 2016).” Kiu et al. then concluded that “interestingly, *netB* was detected in 87.5% JFP isolates (28/32), suggesting that NetB toxin might be involved in haemorrhagic enteritis in dogs and horses.”

This statement and conclusion is incorrect because NetF-producing *C. perfringens* has so far only been shown to be an important cause of canine hemorrhagic gastroenteritis and foal necrotizing enteritis. Several investigations have shown that none of the *netF*-positive *C. perfringens* isolates examined to date encode the *netB* gene (Mehdizadeh Gohari et al., 2015, 2016, 2017). Because of the close homology between NetB and NetE (78% amino acid identity) this is superficially an understandable misinterpretation by Kiu et al. but it is incorrect.

The *netF*+ isolates are clonal in origin, and fall into two clades. Disease in dogs or foals can be associated with either clade (Mehdizadeh Gohari et al., 2017). There is a highly significant epidemiologic association between *netF*-positive strains and fatal enteric disease in foals and dogs (Mehdizadeh Gohari et al., 2015; Busch et al., 2017). A *netF* insertional inactivation mutant has been constructed and shown to be no longer toxic for an equine ovarian (EO) cell line. EO toxicity was restored by complementation *in trans* with the wild-type *netF* gene. These data, together with conjugation and transformation experiments on these plasmids, clearly showed that NetF was responsible for the cytotoxicity for EO cells (Mehdizadeh Gohari et al., 2015).

NetB is a pore-forming toxin produced by *C. perfingens* distinct from though closely related to NetE, and to a lesser extent NetF and other members of the family of beta-sheet pore-forming toxins, with a critical role in the pathogenesis of chicken necrotic enteritis (NE) (Keyburn et al., 2008; Rood et al., 2016). In addition, clonal and host species relationships are evident in NetB-producing *C. perfringens* isolates recovered from chickens with NE (Hibberd et al., 2011; Lepp et al., 2013; Lacey et al., 2015). The statement that the NE isolate NCTC8503 was not found to encode NetB is incorrect since this is a type D, epsilon toxin negative, Australian isolate from 1930, recovered several decades before NE was recognized as a disease of chickens It is not an NE isolate. Given its provenance, it was likely isolated from a sheep with pulpy kidney disease and subsequently lost the epsilon virulence plasmid on laboratory passage. No NE isolates have ever been type D. Kiu et al. in Table 3 reference a 1932 Australian paper for the chicken origin of strain NCTC8503, but this paper discusses only sheep.

The authors suggest that *C. perfringens* has “extreme” pan-genomic variation. However, the species to which it is most analogous in terms of its intestinal and environmental habitat, *Escherichia coli*, has a very similarly sized core genome, 1472 vs. 1470 gene families, and a larger accessory genome (13,296 additional gene families in 61 genomes analyzed) (Lukjancenko et al., 2010) compared to only 10,197 in the Kiu et al *C. perfringens* analysis. Thus describing a species as having extreme variation depends very much to what it is being compared.

Because of the large number of prophages in the *C. perfringens* pan-genome, possibly associated with the lack of CRISPR systems identified by Kiu et al. and the habitat of the species, the authors suggest that diverse prophage-associated gene integration in *C. perfringens* genomes may be central for our understanding of the pathogenicity of *C. perfringens* in terms of human and animal health. Currently, however, there is no evidence that prophage-associated gene integration plays any role in the pathogenicity of *C. perfringens*. No virulence genes have been linked to prophages in *C. perfringens*, unlike *E. coli*. There is long-standing overwhelming evidence however that *tcp* conjugative plasmids are critical to the development and maintenance of virulence in *C. perfringens*, and major advances continue to be made this area (Parreira et al., 2012; Freedman et al., 2015; Watts et al., 2017), including identifying the unique pNetB and pNetF plasmids.

There is currently quite good understanding of the disease-toxinotype link (Theoret and McClane, 2016), although much remains to be done to understand some potential or speculated relationships. We agree with Kiu et al. that WGS studies will be invaluable in teasing out these relationships but painstaking clinical, pathological and epidemiological studies will be critical to further progress.

## Author contributions

All authors listed have made a substantial, direct and intellectual contribution to the work, and approved it for publication.

### Conflict of interest statement

The authors declare that the research was conducted in the absence of any commercial or financial relationships that could be construed as a potential conflict of interest.
